# Propensity score matching is recommended for retrospective comparative analysis of sarcopenia’s clinical prognostic impact on hepatocellular carcinoma resection patients in Eastern and Western cohorts

**DOI:** 10.1097/JS9.0000000000001221

**Published:** 2024-03-04

**Authors:** Jia-Xi Mao, Jing-Jing Li, Yuan-Yu Zhao, Han-Xiang Zhong, Xin-Yi Lu, Li-Ye Zhu, Hong Fu, Guo-Shan Ding, Wen-Yuan Guo, Fei Teng

**Affiliations:** a Department of Liver Surgery and Organ Transplantation, Changzheng Hospital; b Department of Immunology and Medical Immunology State Key Laboratory, Naval Medical University, Shanghai, People’s Republic of China

*Dear Editor*,

Beumer *et al*.^1^ conducted a retrospective study comparing the impact of sarcopenia on the clinical prognosis of patients undergoing hepatocellular carcinoma (HCC) resection in Eastern and Western cohorts, which piqued my interest. The authors used the Netherlands and Japan as representatives of Eastern and Western cohorts, respectively, to investigate the differences in the effect of sarcopenia on overall survival (OS) in postoperative HCC patients between the two regions. The study found that various commonly used cutoff values for sarcopenia had similar predictive abilities, and the impact of sarcopenia on OS in HCC patients was region-dependent, with a stronger correlation observed in the Eastern cohort compared to the Western cohort. This regional comparison of sarcopenia’s effect on the prognosis of tumor patients is relatively rare in published literature and highlights the differences in disease characteristics between these populations. This provides a theoretical basis for future research to consider regional differences. However, there are a few points in the analysis of this article that warrant discussion to enhance its completeness.

Firstly, it can be observed from Table 1^1^ that the Eastern and Western cohorts are not balanced at baseline. To eliminate the influence of confounding factors on the results, it is suggested that propensity score matching (PSM) be used to recompare the perioperative data and prognostic analysis between the two groups.

Secondly, the author did not provide a clear description of the selection criteria for the studies included in Table 2^1^. It is recommended that a comparison of the impact of sarcopenia on HCC patients in Eastern and Western cohorts should be listed in Table 2^1^. However, it should be noted that the studies by Martin *et al*.^2^, Prado *et al*.^3^, Caan *et al*.^4^, Carey *et al*.^5^, Englesbe *et al*.^6^, and Toshima *et al*.^7^ mainly focused on patients with respiratory and gastrointestinal tumors, as well as liver transplant recipients with end-stage liver disease, and none of these studies included HCC patients. If the author intends to include a comparison of the impact of sarcopenia on patients with other types of cancer in Eastern and Western cohorts, the specific cancer types involved should be indicated in the table. For instance, the study conducted by Wu *et al*.^8^ should be contained in Table 2^1^, which analyzes the relationship between sarcopenia measured by Eastern and Western criteria and the prognosis of pancreatic cancer patients. The study found that the occurrence rate of sarcopenia in pancreatic cancer patients calculated by Western criteria was 66.4% (97/146), which was significantly higher than the 11.0% (16/146) calculated by Eastern criteria. There was no significant difference in OS between sarcopenic and non-sarcopenic patients according to Western criteria (*P*=0.807). However, according to Eastern criteria, there was a significant difference (*P*=0.014). This is consistent with the author’s assertion that sarcopenia, as the sole predictive indicator in the OS model, performs better in the Eastern population compared to the Western population. Moreover, the referenced study by Martin *et al*.^2^ did not mention any comparison between Eastern and Western data or 90-day postoperative survival outcomes, so it is unclear where the data in Table 2^1^ came from. In the original study by Caan *et al*.^4^, the percentages of sarcopenia in white, black, Hispanic, and Asian populations were 45.3% (960/2118), 28.2% (66/234), 30.7% (112/365), and 46.0% (239/520), respectively, and the overall percentage of sarcopenic patients was 42%, which did not reach the 63% and 72% mentioned in Table 2^1^ for the West and East groups, respectively. Additionally, the data from Prado *et al*.^3^, Englesbe *et al*.^6^, and Toshima *et al*.^7^ came from a single center each, namely a cancer treatment center serving northern Alberta in Canada, the University of Michigan Medical Center in the U.S., and Kyushu University Hospital in Japan, respectively. These studies did not distinguish between Eastern and Western cohorts. Although Carey *et al*.^5^ included five academic transplant centers including the University of California, San Francisco, University of Pittsburgh, University of Alberta, Mayo Clinic in Arizona, and Cleveland Clinic. They were all located in North America and did not differentiate between Eastern and Western cohorts as well. Therefore, it is recommended to reassess the literature included in Table 2^1^.

Lastly, the titles of Table 2^1^ and Supplementary Table 9^1^ (http://links.lww.com/JS9/A518) are the same, but it seems that the author intends to differentiate between the use of relative cutoff values (percentiles) and absolute cutoff values for the result determination. It would be clearer for readers if these were labeled separately.

We sincerely appreciate the contributions made by the authors to this study and hope that future researchers can further investigate the clinical prognostic impact of sarcopenia on HCC resection patients in Eastern and Western cohorts through meta-analysis and systematic review. We hope that the above revision suggestions can be taken into consideration by the authors to make this retrospective study more comprehensive and rigorous.

## Ethical approval

Not applicable.

## Consent

Not applicable.

## Sources of funding

This research was supported by the National Natural Science Foundation of China (82371792, 81971503), the Open Research Project Program of the State Key Laboratory of Medical Immunology (NKLMI2023K03), Basic medical research project of Naval Medical University (2022QN072), and Clinical technology innovation project of Shanghai Shenkang Hospital Development Center (SHDC12020104).

## Author contribution

J.-X.M.: conceptualization and writing – original draft; J.-J.L.: formal analysis and writing – original draft; Y.-Y.Z.: formal analysis and writing – original draft; H.-X.Z., X.-Y.L., and L.-Y.Z.: investigation and resources; H.F.: supervision; G.-S.D.: supervision; W.-Y.G.: funding acquisition, project administration, and supervision; F.T.: funding acquisition and writing – review and editing.

## Conflicts of interest disclosure

There are no conflicts of interest.

## Research registration unique identifying number (UIN)

Not applicable.

## Guarantor

Fei Teng, e-mail: tengfei@smmu.edu.cn; Wen-Yuan Guo. E-mail: guowenyuan@smmu.edu.cn

## Data availability statement

Not applicable.

## Provenance and peer review

Not commissioned, externally peer-reviewed.

## Supplementary Material
