# Supplementary material for: Propensity score matching is recommended for retrospective comparative analysis of sarcopenia’s clinical prognostic impact on hepatocellular carcinoma resection patients in Eastern and Western cohorts
Source: Int J Surg. 2024 Mar 4;110(5):3116–7. doi: 10.1097/JS9.0000000000001221 (PMC11093475; doi:10.1097/JS9.0000000000001221)
Supplement: Supplementary file 1 [file js9-110-3116-s001.docx]

**Supplementary material**

**Supplementary table 1 – STROBE statement**

|  | | Item No | Recommendation | | Page  No | |
| --- | --- | --- | --- | --- | --- | --- |
| **Title and abstract** | | 1 | (*a*) Indicate the study’s design with a commonly used term in the title or the abstract | | 2 | |
|  |  |  | (*b*) Provide in the abstract an informative and balanced summary of what was done and what was found | | 2 | |
| Introduction | | | | | | |
| Background/rationale | | 2 | Explain the scientific background and rationale for the investigation being reported | | 4 | |
| Objectives | | 3 | State specific objectives, including any prespecified hypotheses | | 5 | |
| Methods | | | | | | |
| Study design | | 4 | Present key elements of study design early in the paper | | 6 | |
| Setting | | 5 | Describe the setting, locations, and relevant dates, including periods of recruitment, exposure, follow-up, and data collection | | 6 | |
| Participants | | 6 | (*a*) *Cohort study*—Give the eligibility criteria, and the sources and methods of selection of participants. Describe methods of follow-up  *Case-control study*—Give the eligibility criteria, and the sources and methods of case ascertainment and control selection. Give the rationale for the choice of cases and controls  *Cross-sectional study*—Give the eligibility criteria, and the sources and methods of selection of participants | | 6 | |
|  |  |  | (*b*) *Cohort study*—For matched studies, give matching criteria and number of exposed and unexposed  *Case-control study*—For matched studies, give matching criteria and the number of controls per case | | *No matching performed* | |
| Variables | | 7 | Clearly define all outcomes, exposures, predictors, potential confounders, and effect modifiers. Give diagnostic criteria, if applicable | | 7 | |
| Data sources/ measurement | | 8* | For each variable of interest, give sources of data and details of methods of assessment (measurement). Describe comparability of assessment methods if there is more than one group | | 7 | |
| Bias | | 9 | Describe any efforts to address potential sources of bias | | 8 | |
| Study size | | 10 | Explain how the study size was arrived at | | 6 | |
| Quantitative variables | | 11 | Explain how quantitative variables were handled in the analyses. If applicable, describe which groupings were chosen and why | | 7 | |
| statistical methods | | 12 | (*a*) Describe all statistical methods, including those used to control for confounding | | 8 | |
|  |  |  | (*b*) Describe any methods used to examine subgroups and interactions | | 8 | |
|  |  |  | (*c*) Explain how missing data were addressed | | 8 | |
|  |  |  | (*d*) *Cohort study*—If applicable, explain how loss to follow-up was addressed  *Case-control study*—If applicable, explain how matching of cases and controls was addressed  *Cross-sectional study*—If applicable, describe analytical methods taking account of sampling strategy | | 8 | |
|  |  |  | (*e*) Describe any sensitivity analyses | | None performed | |
| Results | | | | | |  |
| Participants | 13* | (a) Report numbers of individuals at each stage of study—eg numbers potentially eligible, examined for eligibility, confirmed eligible, included in the study, completing follow-up, and analysed | | 10 | |  |
|  |  | (b) Give reasons for non-participation at each stage | | 10 | |  |
|  |  | (c) Consider use of a flow diagram | | - | |  |
| Descriptive data | 14* | (a) Give characteristics of study participants (eg demographic, clinical, social) and information on exposures and potential confounders | | 10 | |  |
|  |  | (b) Indicate number of participants with missing data for each variable of interest | | 10 | |  |
|  |  | (c) *Cohort study*—Summarise follow-up time (eg, average and total amount) | | 10 | |  |
| Outcome data | 15* | *Cohort study*—Report numbers of outcome events or summary measures over time | | 10 | |  |
|  |  | *Case-control study—*Report numbers in each exposure category, or summary measures of exposure | | *-* | |  |
|  |  | *Cross-sectional study—*Report numbers of outcome events or summary measures | | *-* | |  |
| Main results | 16 | (*a*) Give unadjusted estimates and, if applicable, confounder-adjusted estimates and their precision (eg, 95% confidence interval). Make clear which confounders were adjusted for and why they were included | | 10-12 | |  |
|  |  | (*b*) Report category boundaries when continuous variables were categorized | | - | |  |
|  |  | (*c*) If relevant, consider translating estimates of relative risk into absolute risk for a meaningful time period | | 10-13 | |  |
| Other analyses | 17 | Report other analyses done—eg analyses of subgroups and interactions, and sensitivity analyses | | 11-13 | |  |
| Discussion | | | | | |  |
| Key results | 18 | Summarise key results with reference to study objectives | | 14 | |  |
| Limitations | 19 | Discuss limitations of the study, taking into account sources of potential bias or imprecision. Discuss both direction and magnitude of any potential bias | | 18 | |  |
| Interpretation | 20 | Give a cautious overall interpretation of results considering objectives, limitations, multiplicity of analyses, results from similar studies, and other relevant evidence | | 14-18 | |  |
| Generalisability | 21 | Discuss the generalisability (external validity) of the study results | | 18 | |  |
| Other information | | | | | |  |
| Funding | 22 | Give the source of funding and the role of the funders for the present study and, if applicable, for the original study on which the present article is based | | NA | |  |

*Give information separately for cases and controls in case-control studies and, if applicable, for exposed and unexposed groups in cohort and cross-sectional studies.

**Note:** An Explanation and Elaboration article discusses each checklist item and gives methodological background and published examples of transparent reporting. The STROBE checklist is best used in conjunction with this article (freely available on the Web sites of PLoS Medicine at http://www.plosmedicine.org/, Annals of Internal Medicine at http://www.annals.org/, and Epidemiology at http://www.epidem.com/). Information on the STROBE Initiative is available at www.strobe-statement.org.

**Supplementary table 2 – Sarcopenia cut-off values**

|  | **Cut-off values** |
| --- | --- |
| West | M & BMI < 25 & SMI < 43.6  M & BMI ≥ 25 & SMI < 48.4  F & BMI < 25 & SMI < 35.0  F & BMI ≥ 25 & SMI < 36.9 |
| East | M & BMI < 25 & SMI < 41.8  M & BMI ≥ 25 & SMI < 49.9  F & BMI < 25 & SMI < 33.7  F & BMI ≥ 25 & SMI < 40.2 |

*Legend supplementary table 2 – Specification of the sarcopenia cut-off values for this specific study in the eastern and western cohort. The SMI values are based on the lowest Sex and BMI specific tertile. Abbreviations: Body mass index (BMI); L3-skeletal muscle mass index (SMI); Male (M); Female (F).*

**Supplementary table 3 – Sarcopenia definitions**

|  | **Author (year)** | **Cut-off values** |
| --- | --- | --- |
| Absolute | 1. Martin (2013) | M & BMI < 25 & SMI < 43  M & BMI ≥ 25 & SMI < 53  F & SMI < 41 |
|  | 2. Caan (2017) | M & BMI < 30 & SMI < 52.3  M & BMI ≥ 30 & SMI < 54.3  F & BMI < 30 & SMI < 38.6  F & BMI ≥ 30 & SMI < 46.6 |
|  | 3. Prado (2008) | M & SMI < 52.4  F & SMI < 38.5 |
|  | 4. Carey (2017) | M & SMI < 50  F & SMI < 39 |
| Relative | 5. Engelsbe (2010) | M & SMI < lowest SMI quartile  F & SMI < lowest SMI quartile |
|  | 6. Beumer (2022) | M & BMI < 25 & SMI < lowest SMI tertile  M & BMI ≥ 25 & SMI < lowest SMI tertile  F & BMI < 25 & SMI < lowest SMI tertile  F & BMI ≥ 25 & SMI < lowest SMI tertile |
|  | 7. Toshima (2015) | M & SMI < lowest 5th percentile  F & SMI < lowest 5th percentile |

*Legend supplementary table 3 – Specification of the sarcopenia definitions. Relative sarcopenia definitions were evaluated separately for the eastern and western cohort. Abbreviations: Body mass index (BMI); L3-skeletal muscle mass index (SMI); Male (M); Female (F).*

**Supplementary table 4 – Hepatectomy subtypes**

| **Resection category** | **Hepatectomy subtypes** | **West**  **n (%)** | **East**  **n (%)** | **Resected segments** |
| --- | --- | --- | --- | --- |
| Major resection | Trisectionectomy Right * | 19 (11) | 2 (1) | 6+7+5+8+4 (+1) |
|  | Trisectionectomy Left * | 1 (1) | 3 (1) | 5+8+4+2+3 (+1) |
|  | Hemihepatectomy Right | 44 (25) | 66 (17) | 6+7+5+8 (+1) |
|  | Hemihepatectomy Left | 21 (12) | 58 (15) | 4+2+3 (+1) |
|  | Central bisectionectomy | - | 11 (3) | 5+8+4 |
| Minor resection | Segmentectomy ** | 63 (36) | 51 (13) | 1,2,3,5,6,7,8 |
|  | Sectionectomy ** | - | 146 (39) | 6+7, 5+8, 4, 2+3 |
|  | Wedge | 32 (18) | 42 (11) | Partial segment |

*Legend supplementary table 4: * For the Rotterdam cohort patients were registered as trisectionectomy also if only one additional segment to the hemi hepatectomy was resected. ** For Rotterdam sectionectomy was registered as segmentectomy. Abbreviations – n: number of patients.*

**Supplementary table 5 – Univariable correlation with SMI**

| **Variable** | **n** | **Coef [95%CI]** | **p-value** |
| --- | --- | --- | --- |
| Western center | 553 | 0.08 [-0.01 ; 0.16] | 0.073 |
| Year of surgery | 553 | 0.03 [-0.05 ; 0.12] | 0.443 |
| Male | 553 | 0.51 [0.44 ; 0.57] | <0.001* |
| Age | 553 | -0.17 [-0.25 ; -0.09] | <0.001* |
| BMI | 553 | 0.55 [0.49 ; 0.6] | <0.001* |
| Height | 553 | 0.25 [0.17 ; 0.32] | <0.001* |
| Weight | 553 | 0.53 [0.47 ; 0.59] | <0.001* |
| HCV | 553 | -0.05 [-0.13 ; 0.04] | 0.257 |
| HBV | 529 | 0.06 [-0.03 ; 0.14] | 0.202 |
| TACE | 461 | -0.01 [-0.1 ; 0.08] | 0.774 |
| RFA | 461 | 0.04 [-0.05 ; 0.13] | 0.344 |
| ASA | 548 | 0.05 [-0.03 ; 0.13] | 0.250 |
| Diabetes | 553 | 0.05 [-0.04 ; 0.13] | 0.265 |
| Hypertension | 553 | 0.08 [0.00 ; 0.16] | 0.059 |
| Cardiac comorbidity | 494 | 0.10 [0.02 ; 0.19] | 0.021* |
| Cerebral comorbidity | 494 | -0.03 [-0.12 ; 0.06] | 0.465 |
| Child-Pugh score | 446 | -0.06 [-0.15 ; 0.04] | 0.223 |
| ALBI score | 544 | -0.12 [-0.2 ; -0.04] | 0.005* |
| Tumor number | 553 | -0.09 [-0.17 ; 0.00] | 0.041* |
| Tumor size (cm) | 553 | -0.08 [-0.16 ; 0.00] | 0.059 |
| Microvascular invasion | 545 | -0.06 [-0.15 ; 0.02] | 0.147 |
| Log10(AFP) | 544 | -0.13 [-0.21 ; -0.04] | 0.003* |

*Legend supplementary table 5 – Univariate correlation with SMI. ALBI score and AFP are the last measurement prior to liver transplantation. Abbreviations: Body mass index (BMI); Hepatitis C Virus (HCV); Hepatitis B virus (HBV); Trans arterial chemo embolization (TACE); Radiofrequency ablation (RFA); American Society of Anesthesiologist physical status(ASA); Albumin Bilirubin (ALBI); Alpha Fetoprotein (AFP); L3 Skeletal muscle mass index (SMI).*

**Supplementary table 6 – Effect modification by region**

| **Model** | **n** | **Coef [95%CI]** | **p-value** |
| --- | --- | --- | --- |
| Western center*Year of surgery | 553 | 15.08[-1.91 ; 32.07] | 0.082 |
| Western center*Male | 553 | 1.96[-1.02 ; 4.94] | 0.198 |
| Western center*Age | 553 | 0.19[0.07 ; 0.32] | 0.003* |
| Western center*BMI | 553 | -0.34[-0.65 ; -0.04] | 0.027* |
| Western center*Height | 553 | -9.17[-26.21 ; 7.88] | 0.292 |
| Western center*Weight | 553 | -0.16[-0.24 ; -0.07] | <0.001* |
| Western center*HCV | 553 | 4.81[0.31 ; 9.31] | 0.037* |
| Western center*HBV | 529 | 2.71[-1.29 ; 6.71] | 0.185 |
| Western center*TACE | 461 | -6.81[-14.64 ; 1.01] | 0.089 |
| Western center*RFA | 461 | 4.22[-5.98 ; 14.42] | 0.418 |
| Western center*ASA | 548 | 0.46[-2.22 ; 3.13] | 0.737 |
| Western center*Diabetes | 553 | 4.94[1.65 ; 8.23] | 0.003* |
| Western center*Hypertension | 553 | 3.39[0.33 ; 6.45] | 0.030* |
| Western center*Cardiac comorbidity | 494 | 2.96[-1.42 ; 7.34] | 0.185 |
| Western center*Cerebral comorbidity | 494 | -0.19[-6.05 ; 5.66] | 0.948 |
| Western center*Child-Pugh score | 446 | -1.55[-4.65 ; 1.56] | 0.330 |
| Western center*ALBI score | 544 | 0.34[-2.93 ; 3.62] | 0.837 |
| Western center*Tumor number | 553 | 0.86[-1.38 ; 3.11] | 0.451 |
| Western center*Tumor size | 553 | 0.01[-0.34 ; 0.37] | 0.936 |
| Western center*Microvascular invasion | 545 | 1.74[-1.51 ; 5] | 0.295 |
| Western center*Log_10_(AFP) | 544 | -0.23[-1.33 ; 0.88] | 0.687 |

*Legend supplementary table 6 –Univariate linear regression models explaining L3 Skeletal muscle mass index (SMI) with interaction terms to inspect effect modification of variables between the east and west. Each row represents a new model. Only the data from the interaction terms are shown. ALBI score and AFP are the last measurement prior to liver transplantation. Abbreviations: Body mass index (BMI); Hepatitis C Virus (HCV); Hepatitis B virus (HBV); Trans arterial chemo embolization (TACE); Radiofrequency ablation (RFA); American Society of Anesthesiologist physical status (ASA); Albumin Bilirubin (ALBI); Alpha Fetoprotein (AFP).*

**Supplementary table 7 – Multivariable correlation with SMI**

| **Variable** | **Coef [95%CI]** | **p-value** |
| --- | --- | --- |
| Intercept | 23.73 [10.87 ; 36.58] | 0.001* |
| Western center | -0.45 [-2.24 ; 1.34] | 0.624 |
| Year of surgery (2010,2015] | 1.25 [0 ; 2.49] | 0.05 |
| Year of surgery (2015,2020] | 0.46 [-1.85 ; 2.77] | 0.695 |
| Male | 9.43 [8.03 ; 10.82] | <0.001* |
| Age | -0.13 [-0.19 ; -0.07] | <0.001* |
| BMI | 1.01 [0.85 ; 1.16] | <0.001* |
| HCV | 1.46 [0.01 ; 2.92] | 0.049* |
| HBV | 0.9 [-0.71 ; 2.5] | 0.272 |
| TACE | 0.47 [-1.08 ; 2.02] | 0.553 |
| RFA | 0.23 [-2.04 ; 2.5] | 0.840 |
| ASA | -1.12 [-2.33 ; 0.08] | 0.068 |
| Diabetes | -0.06 [-1.39 ; 1.26] | 0.928 |
| Hypertension | 0.11 [-1.22 ; 1.45] | 0.866 |
| Cardiac comorbidity | 1.29 [-0.46 ; 3.04] | 0.148 |
| Cerebral comorbidity | 0.13 [-1.96 ; 2.21] | 0.906 |
| Child-Pugh score | -0.12 [-1.65 ; 1.4] | 0.873 |
| ALBI score | -0.42 [-2.03 ; 1.2] | 0.613 |
| Tumor number | -0.11 [-0.74 ; 0.51] | 0.720 |
| Tumor size (cm) | -0.09 [-0.25 ; 0.07] | 0.260 |
| Microvascular invasion | 0.2 [-1.19 ; 1.59] | 0.778 |
| Log_10_(AFP) | -0.3 [-0.77 ; 0.18] | 0.221 |

*Legend supplementary table 7 –Multivariable linear regression model, based on 422 observations, explaining L3 Skeletal muscle mass index (SMI). The rows contain information regarding the variables included in a single regression model. ALBI score and AFP are the last measurement prior to liver transplantation. Abbreviations: Body mass index (BMI); Hepatitis C Virus (HCV); Hepatitis B virus (HBV); Trans arterial chemo embolization (TACE); Radiofrequency ablation (RFA); American Society of Anesthesiologist physical status (ASA); Albumin Bilirubin (ALBI); Alpha Fetoprotein (AFP).*

**Supplementary table 8 –Effect modification by region (multivariable)**

| **Model** | **Coef [95%CI]** | **p-value** |
| --- | --- | --- |
| Western center*Year of surgery | -1.78[-4.79 ; 1.23] | 0.246 |
| Western center*Male | -0.72[-3.48 ; 2.04] | 0.608 |
| Western center*Age | 0.10[<0.01 ; 0.21] | 0.047* |
| Western center*BMI | -0.50[-0.79 ; -0.21] | 0.001* |
| Western center*HCV | 6.07[2.69 ; 9.44] | <0.001* |
| Western center*HBV | -0.09[-3.20 ; 3.03] | 0.956 |
| Western center*TACE | 4.02[-2.00 ; 10.03] | 0.192 |
| Western center*RFA | -5.46[-17.08 ; 6.15] | 0.357 |
| Western center*ASA | 0.27[-1.83 ; 2.38] | 0.799 |
| Western center*Diabetes | 1.52[-1.01 ; 4.05] | 0.240 |
| Western center*Hypertension | 0.11[-2.38 ; 2.59] | 0.933 |
| Western center*Cardiac comorbidity | -1.16[-4.41 ; 2.09] | 0.484 |
| Western center*Cerebral comorbidity | -0.97[-5.18 ; 3.25] | 0.653 |
| Western center*Child-Pugh score | 0.22[-2.93 ; 3.37] | 0.890 |
| Western center*ALBI score | 0.78[-2.00 ; 3.57] | 0.581 |
| Western center*Tumor number | -0.37[-2.06 ; 1.32] | 0.670 |
| Western center*Tumor size | -0.03[-0.33 ; 0.27] | 0.837 |
| Western center*Microvascular invasion | 1.31[-1.27 ; 3.90] | 0.320 |
| Western center*Log_10_(AFP) | -0.17[-1.03 ; 0.69] | 0.701 |

*Legend supplementary table 8 –Multivariable linear regression models, based on 422 observations, explaining L3 Skeletal muscle mass index (SMI) with interaction terms to inspect the difference in impact of variables on SMI in an eastern vs western setting given that all other control variables stay the same. Each row represents a new model. Only the data from the interaction terms are shown. Control variables in each model were: Year of surgery, Male gender, Age, BMI, HCV, HBV, TACE, RFA, ASA, Diabetes, Hypertension, Cardiac comorbidity, Cerebral comorbidity, Child-Pugh score, ALBI score, Tumor number, Tumor size, Microvascular invasion and Log_10_(AFP). ALBI score and AFP are the last measurement prior to liver transplantation. Abbreviations: Body mass index (BMI); Hepatitis C Virus (HCV); Hepatitis B virus (HBV); Trans arterial chemo embolization (TACE); Radiofrequency ablation (RFA); American Society of Anesthesiologist physical status (ASA); Albumin Bilirubin (ALBI); Alpha Fetoprotein (AFP).*

**Supplementary table 9 – Predictive performance sarcopenia definitions per region**

| **Sarcopenia definition** | | | **West** | | | | | | **East** | | | | | |
| --- | --- | --- | --- | --- | --- | --- | --- | --- | --- | --- | --- | --- | --- | --- |
| **Author (year)** | **Variables** | **Cut-off** | **Sarcopenia %** | **OS**  **(c-index)** | **RFS**  **(c-index)** | **90d mort (AUC)** | **CD ≥ 3 (AUC)** | **LOS (Deviance)** | **Sarcopenia %** | **OS**  **(c-index)** | **RFS**  **(c-index)** | **90d mort (AUC)** | **CD ≥ 3 (AUC)** | **LOS (Deviance)** |
| Martin (2013) | Gender, BMI | Absolute | 54 | 0.72 | 0.69 | 0.91 | 0.79 | 640.71 | 52 | 0.78 | 0.71 | 0.92 | 0.76 | 2370.33 |
| Caan (2017) | Gender, BMI | Absolute | 63 | 0.72 | 0.69 | 0.91 | 0.79 | 636.66 | 72 | 0.78 | 0.71 | 0.92 | 0.76 | 2356.19 |
| Prado (2008) | Gender | Absolute | 57 | 0.72 | 0.69 | 0.92 | 0.79 | 634.1 | 72 | 0.77 | 0.71 | 0.92 | 0.76 | 2359.77 |
| Carey (2017) | Gender | Absolute | 51 | 0.72 | 0.69 | 0.92 | 0.82 | 622.45 | 63 | 0.78 | 0.71 | 0.91 | 0.77 | 2356.22 |
| Engelsbe (2010) | Gender | Tertile | 33 | 0.72 | 0.7 | 0.91 | 0.8 | 578.11 | 33 | 0.77 | 0.72 | 0.91 | 0.75 | 2376.72 |
| Beumer (2022) | Gender, BMI | Tertile | 33 | 0.72 | 0.7 | 0.9 | 0.79 | 620.22 | 33 | 0.77 | 0.71 | 0.92 | 0.77 | 2365.89 |
| Toshima (2015) | Gender | 5^th^ perc | 19 | 0.72 | 0.69 | 0.9 | 0.78 | 625.51 | 13 | 0.77 | 0.71 | 0.91 | 0.77 | 2380.97 |

*Legend supplementary table 9 – Predictive performance of a multivariable model containing sarcopenia alongside the variables Male gender, Age, Body mass index, Hepatitis C virus infection, Hepatitis B virus infection, Trans arterial chemoembolization, Radiofrequency ablation, American Society of Anesthesiologist physical status, Diabetes, Hypertension, Cardiac comorbidity, Cerebral comorbidity Child-Pugh score, Albumin-bilirubin score, Tumor number, Tumor size in cm, Microvascular invasion, Log_10_(AFP), and Western center. The columns present different outcome measures and the rows various cut-off values to define sarcopenia. Abbreviations: Overall survival (OS); Recurrence free survival (RFS); 90-day mortality (90d mort); Clavien-Dindo score (CD); Length of stay (LOS); Area under the receiver operating characteristic (AUC).*

**Supplementary table 10 – Geographical effect modification of muscle mass on peri-operative outcomes**

|  |  |  |  | **L3-skeletal muscle mass index** | | | **Sarcopenia** | | |
| --- | --- | --- | --- | --- | --- | --- | --- | --- | --- |
| **Outcome** | **Model** | **Cohort** | **n** | **Variable** | **Coef [95%CI]** | **p-value** | **Variable** | **Coef [95%CI]** | **p-value** |
| 90d | Univariable | All | 553 | SMI | -0.02[-0.07 ; 0.02] | 0.285 | Sarcopenia | 0.53[-0.21 ; 1.26] | 0.152 |
|  | Univariable | West | 174 | SMI | -0.03[-0.09 ; 0.03] | 0.340 | Sarcopenia | 0.2[-0.92 ; 1.25] | 0.711 |
|  | Univariable | East | 379 | SMI | -0.03[-0.09 ; 0.04] | 0.433 | Sarcopenia | 0.87[-0.18 ; 1.94] | 0.101 |
|  | Univariable (interaction) | All | 553 | SMI | -0.03[-0.09 ; 0.04] | 0.433 | Sarcopenia | 0.87[-0.18 ; 1.94] | 0.101 |
|  |  |  |  | SMI*Western center | 0.00[-0.09 ; 0.08] | 0.937 | Sarcopenia* Western center | -0.67[-2.19 ; 0.81] | 0.380 |
|  | Multivariable | All | 422 | SMI | 0.02[-0.06 ; 0.11] | 0.541 | Sarcopenia | 0.02[-1.00 ; 1.00] | 0.968 |
|  | Multivariable | West | 144 | SMI | -0.01[-0.15 ; 0.13] | 0.836 | Sarcopenia | -0.42[-2.13 ; 1.12] | 0.606 |
|  | Multivariable | East | 278 | SMI | 0.04[-0.1 ; 0.17] | 0.559 | Sarcopenia | 0.61[-1.09 ; 2.38] | 0.482 |
|  | Multivariable (interaction) | All | 422 | SMI | 0.03[-0.06 ; 0.13] | 0.466 | Sarcopenia | 0.39[-0.98 ; 1.77] | 0.569 |
|  |  |  |  | SMI* Western center | -0.02[-0.14 ; 0.09] | 0.697 | Sarcopenia* Western center | -0.78[-2.79 ; 1.14] | 0.430 |
| CD ≥ 3 | Univariable | All | 548 | SMI | -0.03[-0.06 ; -0.01] | 0.019* | Sarcopenia | 0.88[0.43 ; 1.34] | <0.001* |
|  | Univariable | West | 169 | SMI | -0.05[-0.09 ; -0.01] | 0.019* | Sarcopenia | 0.94[0.24 ; 1.66] | 0.009* |
|  | Univariable | East | 379 | SMI | -0.02[-0.06 ; 0.01] | 0.208 | Sarcopenia | 0.84[0.24 ; 1.45] | 0.006* |
|  | Univariable (interaction) | All | 548 | SMI | -0.02[-0.06 ; 0.01] | 0.208 | Sarcopenia | 0.84[0.24 ; 1.45] | 0.006* |
|  |  |  |  | SMI* Western center | -0.03[-0.08 ; 0.03] | 0.369 | Sarcopenia* Western center | 0.10[-0.83 ; 1.04] | 0.832 |
|  | Multivariable | All | 419 | SMI | -0.05[-0.10 ; <-0.01] | 0.053 | Sarcopenia | 0.79[0.21 ; 1.37] | 0.007* |
|  | Multivariable | West | 141 | SMI | -0.07[-0.14 ; 0.01] | 0.087 | Sarcopenia | 0.91[-0.03 ; 1.86] | 0.057 |
|  | Multivariable | East | 278 | SMI | -0.05[-0.12 ; 0.02] | 0.181 | Sarcopenia | 0.76[-0.03 ; 1.56] | 0.059 |
|  | Multivariable (interaction) | All | 419 | SMI | -0.03[-0.09 ; 0.02] | 0.269 | Sarcopenia | 0.68[-0.07 ; 1.43] | 0.076 |
|  |  |  |  | SMI* Western center | -0.04[-0.11 ; 0.03] | 0.307 | Sarcopenia* Western center | 0.27[-0.86 ; 1.41] | 0.642 |
| LOS | Univariable | All | 552 | SMI | -0.01[-0.01 ; 0.00] | <0.001* | Sarcopenia | 0.19[0.15 ; 0.23] | <0.001* |
|  | Univariable | West | 173 | SMI | -0.02[-0.02 ; -0.01] | <0.001* | Sarcopenia | 0.36[0.27 ; 0.45] | <0.001* |
|  | Univariable | East | 379 | SMI | 0.00 [0.00 ; 0.00] | 0.374 | Sarcopenia | 0.15[0.1 ; 0.19] | <0.001* |
|  | Univariable (interaction) | All | 552 | SMI | 0.00[0.00 ; 0.00] | 0.374 | Sarcopenia | 0.15[0.1 ; 0.19] | <0.001* |
|  |  |  | 552 | SMI* Western center | -0.02[-0.02 ; -0.01] | <0.001* | Sarcopenia* Western center | 0.22[0.12 ; 0.32] | <0.001* |
|  | Multivariable | All | 422 | SMI | -0.01[-0.02 ; -0.01] | <0.001* | Sarcopenia | 0.15[0.11 ; 0.2] | <0.001* |
|  | Multivariable | West | 144 | SMI | -0.02[-0.03 ; -0.01] | <0.001* | Sarcopenia | 0.27[0.16 ; 0.38] | <0.001* |
|  | Multivariable | East | 278 | SMI | -0.01[-0.01 ; 0.00] | <0.001* | Sarcopenia | 0.11[0.05 ; 0.16] | <0.001* |
|  | Multivariable (interaction) | All | 422 | SMI | -0.01[-0.01 ; 0.00] | <0.001* | Sarcopenia | 0.12[0.06 ; 0.17] | <0.001* |
|  |  |  | 422 | SMI* Western center | -0.02 [-0.03 ; -0.01] | <0.001* | Sarcopenia*West | 0.19[0.07 ; 0.3] | 0.001* |

*Legend supplementary table 10 – Impact of the L3-skeletal muscle mass index (SMI) and Sarcopenia on the outcomes 90-day mortality (90d); Clavien-Dindo score (CD); and Length of hospital stay (LOS). Each row describes on the left a model for SMI, and on the right a model for Sarcopenia. Only the coefficients of the variables SMI, Sarcopenia, and the interaction term with Western center are shown. Control variables in the multivariable models consisted of: Male gender, Age, BMI, Hepatitis C virus (HCV), Hepatitis B virus (HBV), Trans-arterial chemoembolization (TACE) , Radiofrequency ablation (RFA), American Society of Anesthesiologist physical status (ASA), Diabetes, Hypertension, Cardiac comorbidity, Cerebral comorbidity, Child-Pugh score, Albumin-Bilirubin (ALBI) score, Tumor number at radiology, Tumor size in cm at radiology, Microvascular invasion, and log_10_ Alpha fetoprotein (AFP). Note: Point estimates and confidence intervals on the odds ratio scale can be obtained for 90-day survival and severe complications by taking the exponent.*
